# La-doped MIL-88B(Fe)–NH_2_: a mixed-metal–organic framework photocatalyst for highly efficient reduction of Cr(vi) in an aqueous solution

**DOI:** 10.1039/d4ra03351e

**Published:** 2024-06-27

**Authors:** Minh Hue Dang Thi, Linh Giang Hoang Thi, Chinh Dang Huynh, Hoai Phuong Nguyen Thi, Duc D. La

**Affiliations:** a School of Chemistry and Life Sciences, Hanoi University of Science and Technology Hanoi Vietnam; b Department of Chemistry and Environment, Joint Vietnam-Russia Tropical Science and Technology Research Center 63 Nguyen Văn Huyen Street, Cau Giay Ha Noi Vietnam; c Laboratory of Advanced Materials Chemistry, Institute for Advanced Study in Technology, Ton Duc Thang University Ho Chi Minh City Vietnam laducduong@tdtu.edu.vn; d Faculty of Applied Sciences, Ton Duc Thang University Ho Chi Minh City Vietnam

## Abstract

With the aim to resolve the problem of water pollution, we herein propose a new photocatalyst based on metal–organic frameworks (MOFs), called La-doped MIL-88B(Fe)–NH_2_ (MIL-88B((1 − *x*)Fe/*x*La)-NH_2_), which was designed and employed for the photocatalytic reduction of Cr(vi) in aqueous solutions. MIL-88B((1−*x*)Fe/*x*La)-NH_2_ materials with different *x* values were synthesized *via* a one-pot solvothermal method. Their characteristics were investigated using various techniques, including X-ray diffraction (XRD), scanning electron microscopy (SEM), energy-dispersive spectroscopy (EDS), Brunauer–Emmett–Teller (BET) analysis, Fourier-transform infrared (FT-IR) spectroscopy and ultraviolet-visible diffuse reflectance spectroscopy (UV-vis DRS). We found that compared to pristine MIL-88B(Fe)–NH_2_ with a photocatalytic efficiency of 67.08, MIL-88B((1 − *x*)Fe/*x*La)-NH_2_ materials with *x* = 0.010, 0.025 and 0.050 exhibit excellent photocatalytic efficiencies reaching 88.21, 81.19 and 80.26%, respectively, after only 30 minutes of irradiation at a small catalyst dosage of 0.2 g L^−1^. These La-doped MIL-88B(Fe)–NH_2_ photocatalysts can work well under mild conditions (pH = 6). Furthermore, they are robust—can be recycled for at least four consecutive runs without any activity loss. This novel material is promising for the photocatalytic degradation of pollutants.

## Abstract 2

A novel method to fabricate La-doped MIL-88B(Fe)–NH_2_ mixed-metal–organic frameworks is reported. The prepared rare earth-doped MOFs show remarkable photocatalytic performance for the removal of Cr(vi) in aqueous media.

## Graphical abstract

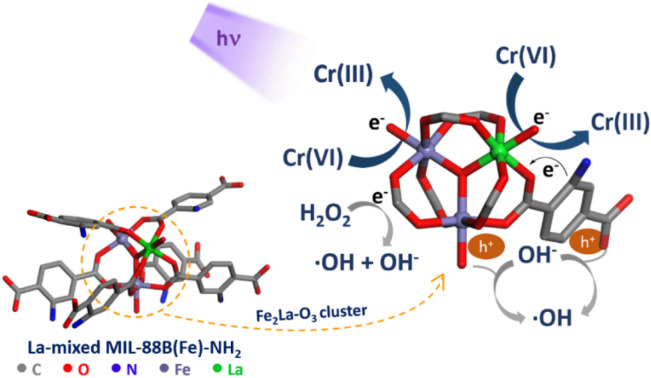

## Introduction

The increase in toxic heavy metal ion contamination in aquatic environments has become a serious issue worldwide. Hexavalent chromium (Cr(vi)) is a typical contaminant that is widely used in various industrial fields such as electroplating, leather tanning, cooling tower blowdown, and rinse waters.^1^ Waste Cr(vi) compounds are discharged easily into water bodies and cause serious harm to human health and organisms.^2^ Alternatively, Cr(iii) is an essential trace metal involved in protein structure stabilization and glucose and lipid metabolism.^3^ Therefore, reducing Cr(vi) to Cr(iii) is considered an effective way for Cr(vi) removal from water.

A number of methods, including chemical, electrochemical and biological processes, are applied to aqueous Cr(vi) reduction.^4^ Therein, the reduction of Cr(vi) to Cr(iii) *via* a photocatalysis process is a fruitful method. This photocatalytic technique is based on the electron/hole (e^−^/h^+^) pairs generated in semiconductor materials under light illumination whose photon energy is greater than the semiconductor's bandgap energy.^5^ Many studies have reported the photocatalytic reduction of Cr(vi) over TiO_2_, which is the most widely studied photocatalyst.^6–8^ However, its catalytic efficiency is limited by its large bandgap energy (3.2 eV) and the high recombination rate of photogenerated e^−^/h^+^ pairs.^9^

Metal–organic frameworks (MOFs) are a class of porous and crystalline materials composed of metal ions/ion clusters and organic ligands. Large specific surface area, structural tunability and reversible adsorption are the outstanding features of MOFs.^10^ As a result, MOFs can be applied to a series of applications such as catalysis,^11,12^ gas storage and separation,^13,14^ cell imaging,^15^ and sensing.^16^ In the catalysis area, in particular photocatalysis, MOFs have become dominant photocatalysts for treating water pollution because of their low e^−^/h^+^ recombination probability due to ligand-to-metal charge transfer (LMCT).^17,18^ Fe-based MOFs (Fe-MOFs) are a family of potential materials in this field owing to their relatively small bandgap in the range of 1.6–2.8 eV,^11,12,19–22^ low toxicity and intrinsic stability.^23^ Moreover, Fe-MOFs contain unsaturated iron(iii) ions with high catalytic activity, and this ensures their catalytic ability in advanced oxidation processes (AOPs), in particular Fenton-like processes.^23^ Among them, MIL-88B(Fe)–NH_2_ (MIL: Materials of Institute Lavoisier) is a common Fe-MOF material whose structure is built up by trimers of iron(iii) octahedra and 2-aminoterephthalate ligands.^24^ Compared to other MOFs, MIL-88B(Fe)–NH_2_ exhibits high catalytic ability,^25^ chemical stability, structural flexibility, and abundant raw sources.^16^ Hence, it attracts remarkable attention in a wide range of applications such as heterogeneous catalysis,^26^ adsorption,^27^ sensing^16^ and batteries.^28^

Many strategies have been used in order to enhance the photocatalytic efficiency of MOFs as well as other semiconductors. In this aspect, mixing rare earth elements (REEs) (La, Ce, Sb and so on) with these materials has been proven to be a feasible solution.^29–31^ Opposite to d-block metals, REE-metals have unique electronic properties because of their 4f electron configurations that are shielded from outermost subshell 5s and 5p, and REE-metals have distinct electronic and magnetic properties that are not significantly altered by coordinating ligands. Furthermore, REEs in general and lanthanum (_57_La) in particular are able to act as electron traps thanks to a plenty of empty orbitals in 4f and 5d subshells, thereby slowing down the e^−^/h^+^ recombination rate and consequently improving the efficiency. The application of the Lanthanum-MOFs has been reported in various fields of catalysis, adsorption of toxic and heavy metal ions, and sensing. Further modification of La–Fe MOFs can improve the surface area and catalytic capability of the materials. In this work, we aimed to synthesize and apply La-doped MIL-88B(Fe)–NH_2_ for the photocatalytic removal of Cr(vi). Various methods including X-ray diffraction (XRD), scanning electron microscopy (SEM), energy-dispersive spectroscopy (EDS), Brunauer–Emmett–Teller (BET) analysis, Fourier-transform infrared (FT-IR) spectroscopy, and ultraviolet-visible diffuse reflectance spectroscopy (UV-vis DRS) were employed to characterize the as-synthesized photocatalysts, and ultraviolet-visible (UV-vis) spectroscopy was used to determine the remaining Cr(vi) concentration in aqueous media. We found that the introduction of lanthanum(iii) into the MIL-88B(Fe)–NH_2_ structure enhances the efficiency of Cr(vi) removal. Besides, experiments with different lanthanum(iii) contents were conducted to find out the influence of the mixed lanthanum(iii) content on the efficiency of the Cr(vi) photoreduction.

## Results and discussion

### Characterizations of materials

**Fig. 1:**
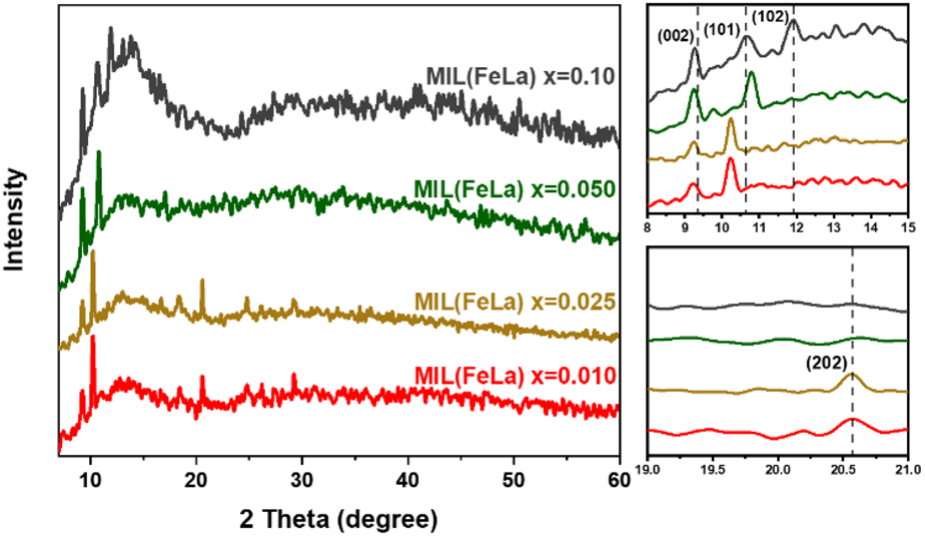
XRD spectra of La-doped MIL-88B(Fe)–NH_2_ materials (MIL(FeLa)) under the solvothermal condition: 150 °C and 12 hours.

Fig. 1 shows the XRD spectra of MIL-88B((1 − *x*)Fe/*x*La)-NH_2_ (*x* = 0.010, 0.025, 0.050 and 0.10) materials (abbreviated in the graph: MIL(FeLa)) under the solvothermal condition of 150 °C and 12 hours. As reported in our previous work on MIL-88B(Fe)–NH_2_,^16^ two characteristic peaks of the MIL-88B(Fe)–NH_2_ phase appeared at 2*θ* ≈ 9.3 and 10.6° corresponding to the (002) and (101) lattices (CCDC 647646). Two of these diffraction peaks also appear for the MIL-88B((1 − *x*)Fe/*x*La)-NH_2_ materials, but they record a slight variation. In particular, the peak of the (002) lattice moves to a smaller angular position on the XRD pattern of all La^3+^ ratios (2*θ* ≈ 9.2°). With the (101) lattice, the peak shifts to the position 2*θ* ≈ 10.3° in the samples with *x* = 0.010 and 0.025, 2*θ* ≈ 10.8° in the sample with *x* = 0.050. More characteristic peaks are observed at 2*θ* ≈ 11.9° (sample with *x* = 0.10) and 2*θ* ≈ 20.6° (samples with *x* = 0.010 and 0.025) corresponding to the (102) and (202) lattice surfaces of MIL-88B(Fe)–NH_2_ (CCDC 647646). These shifts as well as the appearance of additional peaks on the XRD spectra of the La-doped MIL-88B(Fe)–NH_2_ material are the result of the presence of La^3+^ in the MIL-88B(Fe)–NH_2_ structure.^32^ Importantly, no impure phases exist in the MIL-88B((1 − *x*)Fe/*x*La)-NH_2_ (*x* = 0.010, 0.025 and 0.050) spectra, demonstrating the single-phase material. By contrast, the obtained MIL-88B((1 − *x*)Fe/*x*La)-NH_2_ (*x* = 0.10) material is impure, as proven by the appearance of strange peaks in the range 2*θ* ≈ 12.5–15.0°. It could be the result of ligand competition between La^3+^ and Fe^3+^, or/and the formation of other lanthanum compounds. The ligand competition between La^3+^ and Fe^3+^ could lead to the formation of separate La-based MOFs. Many previous studies about MOF materials based on rare earth elements revealed that their MOF structure is complex and consequently difficult to determine because they are mostly built by metal ion chains with a large coordination number (usually 9).^33^ Besides, some La(iii) compounds could form during the reaction such as La(NO_3_)_3_,^34^ LaCl_3_,^35^ LaClO and La(OH)_3_.^36^ These findings revealed that MIL-88B((1 − *x*)Fe/*x*La)-NH_2_ was successfully synthesized at *x* values equal to 0.010, 0.025 and 0.050.

**Fig. 2:**
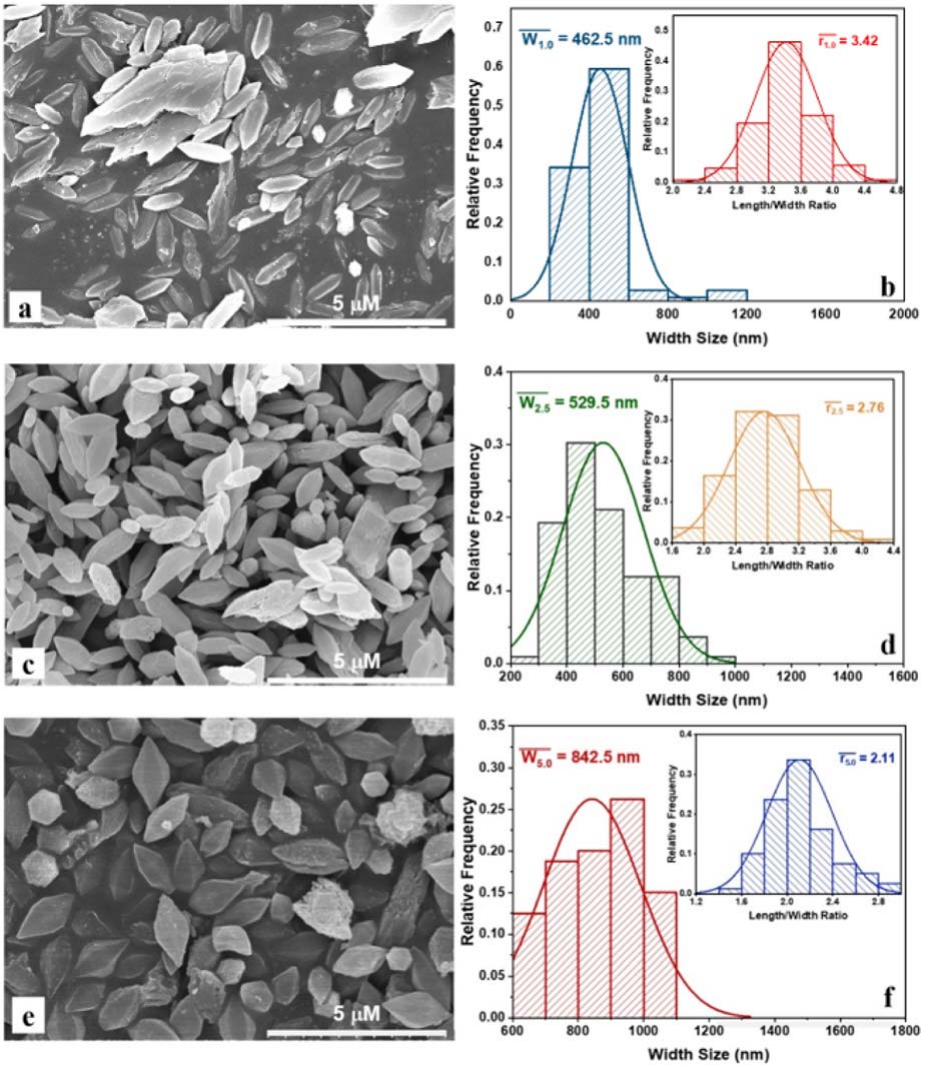
SEM images and particle size distribution of MIL-88B((1 − *x*)Fe/*x*La)-NH_2_ with *x* = 0.010 (a and b), *x* = 0.025 (c and d) and *x* = 0.050 (e and f).

Morphology and particle size distribution of MIL-88B((1 − *x*)Fe/*x*La)-NH_2_ (*x* = 0.010, 0.025 and 0.050) materials are presented in Fig. 2. The material particles show a bipyramidal hexagonal prism shape, similar to previous works on MIL-88B(Fe)–NH_2_.^16,19,37–39^ Furthermore, a relatively uniform distribution of the particles in all materials is observed. The size distribution and mean size were calculated using the ImageJ software, and the outcomes record changes in the particle size in the obtained MOFs. The average widths of MIL-88B((1 − *x*)Fe/*x*La)-NH_2_ at *x* = 0.010, 0.025 and 0.050 are 462.5, 529.5 and 842.5 nm, respectively; corresponding to the average length/width ratio of 3.42; 2.76 and 2.11 (Table 1). It can be seen that the width size is proportional to the La^3+^ content, whereas the trend of the length/width ratio is opposite. The large radius of the La^3+^ ion compared to Fe^3+^ and the structural swell may cause this change.^32^

**Table 1:** Average particle sizes of MIL-88B((1 − *x*)Fe/*x*La)-NH_2_ materials

Average values	MIL-88B((1 − *x*)Fe/*x*La)-NH_2_ (*x* = 0.010)	MIL-88B((1 − *x*)Fe/*x*La)-NH_2_ (*x* = 0.025)	MIL-88B((1 − *x*)Fe/*x*La)-NH_2_ (*x* = 0.050)
Width (nm)	462.5	529.5	842.5
Length/width ratio	3.42	2.76	2.11

**Fig. 3:**
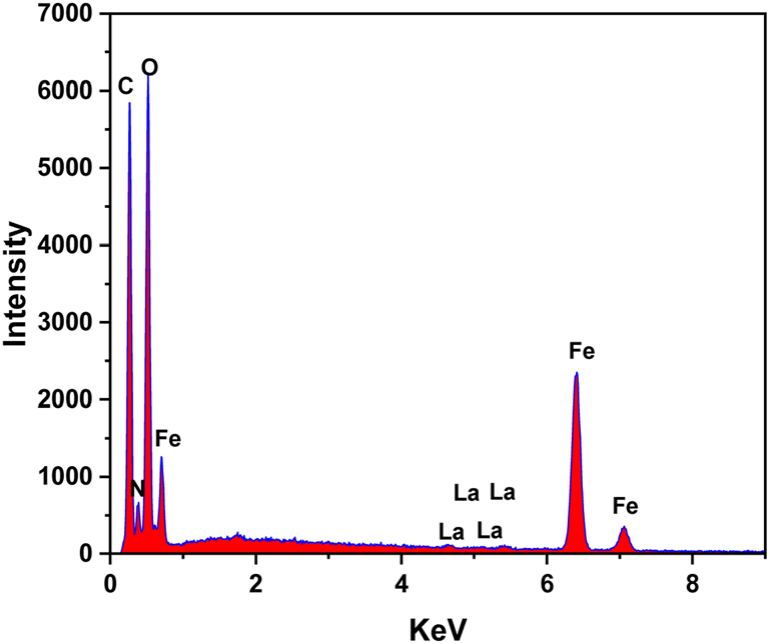
EDS spectrum of MIL-88B((1 − *x*)Fe/*x*La)-NH_2_ (*x* = 0.010).

The EDS analysis technique was employed to determine the element compositions of MIL-88B((1 − *x*)Fe/*x*La)-NH_2_ (*x* = 0.010), as shown in Fig. 3. The results indicate the presence of La elements in the MIL-88B(Fe)–NH_2_ structure.

**Fig. 4:**
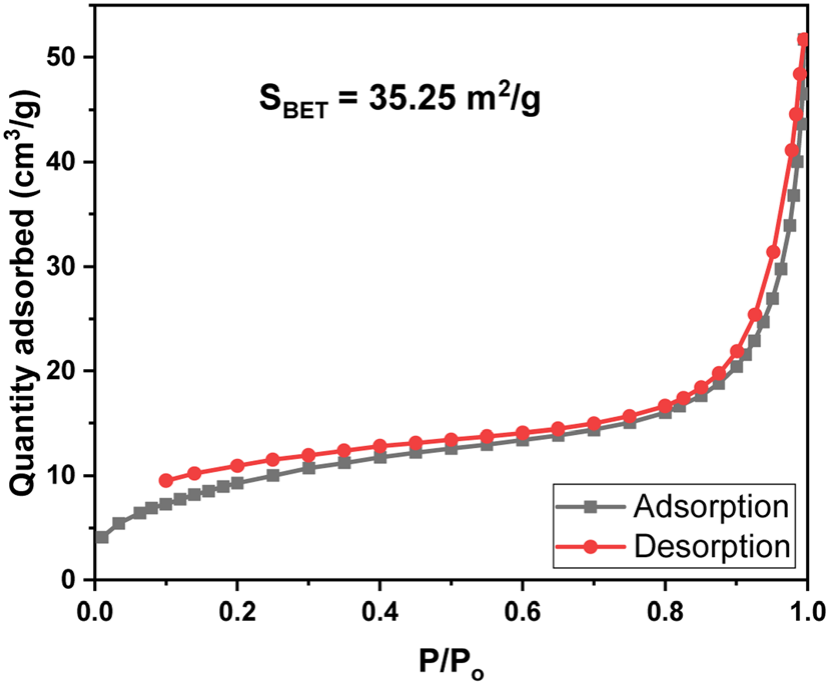
N_2_ adsorption–desorption isotherms of MIL-88B((1 − *x*)Fe/*x*La)-NH_2_ (*x* = 0.010).

The N_2_ adsorption–desorption isotherm analysis (BET) of MIL-88B((1 − *x*)Fe/*x*La)-NH_2_ (*x* = 0.010) is shown in Fig. 4. It can be seen that the N_2_ adsorption–desorption curve of MIL-88B((1 − *x*)Fe/*x*La)-NH_2_ (*x* = 0.010) displayed type IV isotherms with hysteresis corresponding to capillary condensation, which is typical of mesoporous materials. The measured surface area from BET analysis (*S*_BET_) of MIL-88B((1 − *x*)Fe/*x*La)-NH_2_ (*x* = 0.010) is 35.3 m^2^ g^−1^ (Fig. 4). Normally, the *S*_BET_ values of Fe-MOF materials are lower than those of other MOF families^40,41^ due to their closed micropore structure (Table 2). Micropores in the Fe-MOFs' structure are incompatible with N_2_ in terms of size, thereby restricting N_2_ adsorption.^45,46^ Besides, the surface area of Fe-MOFs is affected by different synthesis conditions and methods as well.

**Table 2:** Comparison of the BET surface area of MIL-88B((1 − *x*)Fe/*x*La)-NH_2_ (*x* = 0.010) with other Fe-MOFs

Material	Synthesis method (solvent)	*S*_BET_ (m^2^ g^−1^)	References
MIL-53(Fe)	Solvothermal	6.48	42
MIL-53(Fe)	Solvothermal	9.77	43
MIL-88B(Fe)–NH_2_	Solvothermal	19.2	44
MIL-88B(Fe)–NH_2_	Solvothermal (H_2_O/ethanol)	2.35	43
MIL-88B(Fe)–NH_2_	Solvothermal (DMF/ethanol)	8.9	42
MIL-88B(Fe)–NH_2_	Solvothermal (DMF)	13.43	16
MIL-88B((1 − *x*)Fe/*x*La)-NH_2_ (*x* = 0.010)	Solvothermal (DMF)	35.25	This work

**Fig. 5:**
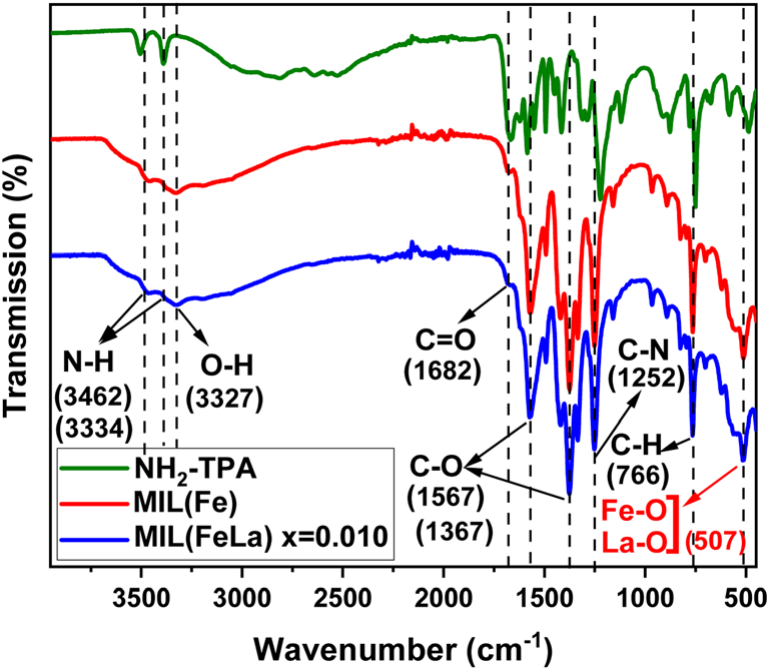
FT-IR spectra of NH_2_-TPA, MIL-88B(Fe)–NH_2_ and MIL-88B((1 − *x*)Fe/*x*La)-NH_2_ (*x* = 0.010).

Fig. 5 shows infrared spectra of the NH_2_-TPA ligand, MIL-88B(Fe)–NH_2_ and MIL-88B((1 − *x*)Fe/*x*La)-NH_2_ (*x* = 0.010) materials. Two peaks at 3462 and 3334 cm^−1^ are attributed to the asymmetric and symmetric stretching vibrations of N–H bonds, respectively. Similarly, two peaks appear at 1567 and 1367 cm^−1^ due to the asymmetric and symmetric C–O stretching oscillation. A peak at 1682 cm^−1^ represents the presence of the C=O group. The peaks at 1252 cm^−1^ and 766 cm^−1^ correspond to C_sp_^2^–N and C_sp_^2^–H bending vibrations. All of these summits are observed in the infrared graph of the TPA-NH_2_ ligand and the as-synthesized MOFs. Additionally, in the spectrum of MIL-88B(Fe)–NH_2_ and MIL-88B((1 − *x*)Fe/*x*La)-NH_2_ (*x* = 0.010), there appear other peaks which characterize new binding vibrations. Particularly, the peak appears at 3327 cm^−1^ due to the presence of O–H vibration that belongs to H_2_O molecules adsorbed in the MOF material. The characteristic vibrations of Fe–O and La–O bonds are observed at 507 cm^−1^, evidence of the binding formation among Fe^3+^, La^3+^ and the COO– groups in the ligand. As such, the FT-IR results contribute to confirming the bond formation of the metal centers with the ligand as well as the structural stability when induced by the La^3+^ ion.

**Fig. 6:**
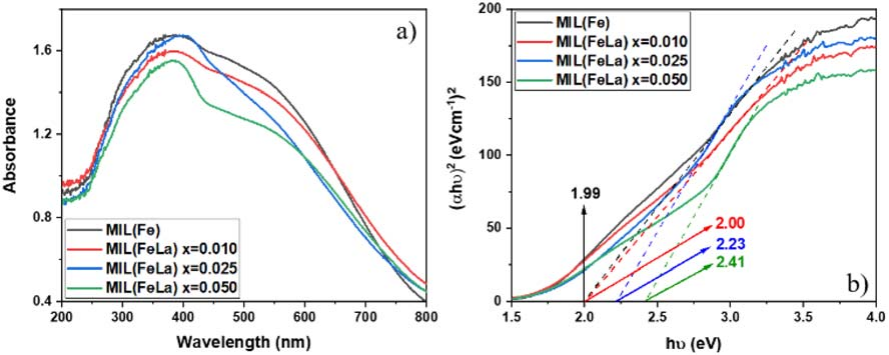
UV-vis-DRS spectra (a) and Tauc plots (b) of MIL-88B(Fe)–NH_2_ and MIL-88B((1 − *x*)Fe/*x*La)-NH_2_ (*x* = 0.010, 0.025 and 0.050).

Fig. 6a shows the UV-vis DRS results of MIL-88B(Fe)–NH_2_ and MIL-88B((1 − *x*)Fe/*x*La)-NH_2_ (*x* = 0.010, 0.025 and 0.050) materials. Compared to the original MIL-88B(Fe)–NH_2_ material, the wavelength at which the maximum absorption of MIL-88B((1 − *x*)Fe/*x*La)-NH_2_ materials takes place does not change significantly, but there is difference in absorption intensity. MIL-88B(Fe)–NH_2_ has the maximum wavelength (*λ*_max_) at 390 nm with an absorption edge extending to the visible light region while the absorbance in the spectra of the MIL-88B((1 − *x*)Fe/*x*La)-NH_2_ (*x* = 0.010, 0.025 and 0.050) drops slightly.

The bandgap energies of MIL-88B(Fe)–NH_2_ and MIL-88B((1 − *x*)Fe/*x*La)-NH_2_ (*x* = 0.010, 0.025 and 0.050) photocatalysts were determined using the Kubelka–Munk equation and the Tauc plot^47^ as follows:(*αhν*)^2^ = *A*(*hν* – *E*_g_)where *α* is the absorption coefficient, *h* is Planck's constant, *ν* is the photon's frequency, *A* is a proportionality constant and *E*_g_ is the bandgap energy.

As shown in Fig. 6b, the bandgap energy (*E*_g_) increases from 1.99, 2.00, 2.23 and 2.41 eV corresponding to the *x* value increasing from 0 (MIL-88B(Fe)–NH_2_) to 0.050. It can be seen that La^3+^ inserted into the MIL-88B(Fe)–NH_2_ structure expands the bandgap energy, and this energy increases proportionally to the La^3+^ content. This widening can be explained by the Burstein–Moss effect.^48^ La^3+^ tends to contribute more electrons than Fe^3+^ because the large ionic radius of La^3+^ reduces the electrostatic interaction between the outer electrons and the nucleus, leading to the Fermi level being filled with electrons. Therefore, the following excited electrons can only move to an energy state higher than the Fermi level, causing the bandgap expansion. Moreover, crystal defects can be a factor that makes the *E*_g_ value shift, and a decrease in crystal defects results in the *E*_g_ increase.^49^ The binding energy of La–O is stronger than that of Fe–O (*E*_La–O_ = 798 kJ mol^−1^ > *E*_Fe–O_ = 407 kJ mol^−1^ (ref. 50)) which contributes to reducing the number of defects in the crystal lattice.

### Photocatalytic study

**Fig. 7:**
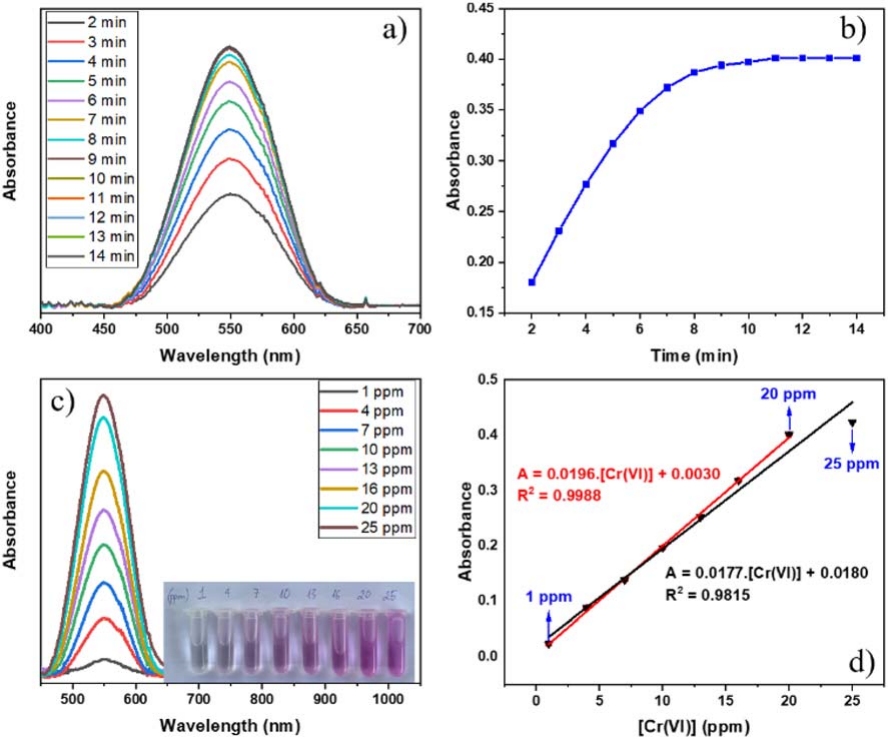
(a) Adsorption spectra and (b) time-absorbance line graph of the Cr–DPC complex. (c) Adsorption spectra of Cr–DPC complex solutions with different initial Cr(vi) concentrations and (d) linear relationship between the absorbance at 550 nm and Cr(vi) concentration.

The results of UV-vis spectra of the Cr–DPC complex solution at various reaction intervals in the range of 2–14 minutes and its time-absorbance line graph are indicated in Fig. 7a and b. The output reveals that the maximum absorbance reaches a wavelength of 550 nm (*λ*_max_ = 550 nm),^51^ and the absorbance of the Cr–DPC solution remains stable after 11 minutes. Therefore, the absorbance of the following Cr–DPC solutions is measured at *λ*_max_ = 550 nm after 11 minutes of reaction. To determine the linear range between the Cr(vi) concentration and the absorbance, we built two calibration curves in the Cr(vi) concentration range of 1–25 ppm (Fig. 7c and d). Linearity is observed from 1 ppm to 20 ppm using equation *A* = 0.0196[Cr(vi)] + 0.0030 (*R*^2^ = 0.9988).

**Fig. 8:**
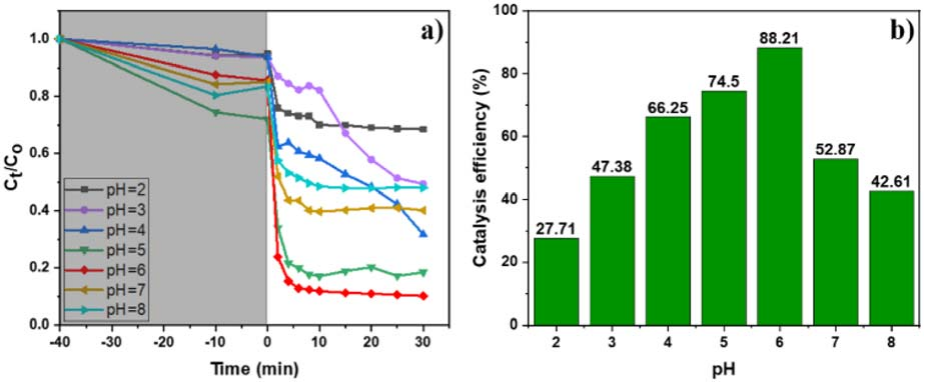
Effect of pH on the photocatalytic performance of MIL-88B((1 − *x*)Fe/*x*La)-NH_2_ (*x* = 0.010) (initial Cr(vi) concentration: 20 ppm; catalyst dosage: 0.2 g L; H_2_O_2_: 3% (1 mL L^−1^); UV irradiation): (a) Cr(vi) removal efficiency at different time and pH solution and (b) Cr(vi) removal efficiencies at different pH solutions.

Previous studies have reported that the pH environment has considerable effects on the Cr(vi) photoreduction ability.^11,52,53^ Under a basic condition, the existing form of Cr(OH)_3_ precipitation of Cr(iii) can cover active sites on the catalyst surface leading to a reduction in the efficiency. Therefore, our work focused on evaluating the pH influence on the catalytic activity for the photoreduction of Cr(vi) to Cr(iii) using the MIL-88B((1 − *x*)Fe/*x*La)-NH_2_ (*x* = 0.010) material under acidic, neutral and weak alkaline conditions, pH = 2–8 (Fig. 8). It can be seen that the weak acid environment is suitable for Cr(vi) reduction, and the best efficiency reaches 88.21% at pH = 6. The efficiency drops significantly when the pH increases, remaining at about 53% at pH = 7 and 43% at pH = 8. The same trend is observed when the pH value is below 6: the lower the pH, the poorer the performance.

Different existing species of Cr(vi) and Cr(iii) may affect the material's catalytic efficiency. In the pH range from 2 to 6, Cr(vi) exists in Cr_2_O_7_^2−^ and HCrO_4_^−^ forms, and Cr(iii) exists in [Cr(H_2_O)_6_]^3+^, Cr(OH)_2_^+^, Cr(OH)^2+^ and Cr(OH)_3_ colloid forms.^54^ Meanwhile, in the neutral and weak alkaline environment (6 < pH < 8), CrO_4_^2−^ and Cr(OH)_3_ solids are the main forms of Cr(vi) and Cr(iii), respectively.^54^ Under neutral and weak alkaline conditions, the CrO_4_^2−^ form of Cr(vi) can inhibit the reduction process due to the low redox potential of CrO_4_^2−^/Cr(OH)_3_ (*E*^o^ −0,13 V).^55^ Besides, the Cr(OH)_3_ solid can mask active sites, leading to the limitation of the material's catalytic activity. In the acidic environment, the lower the pH, the higher the redox potential of Cr(vi)/Cr(iii) (*E*_Cr(VI)/Cr(III)_), which is beneficial for the reduction of Cr(vi) to Cr(iii). Nonetheless, the results show the achieved highest performance at pH = 6. Thus, the Cr(vi) conversion performance can be affected by a Fenton-like process. Fe^3+^ ions in the structure can react with H_2_O_2_, which increases the number of Fe^2+^ ions *via* the Fenton mechanism 
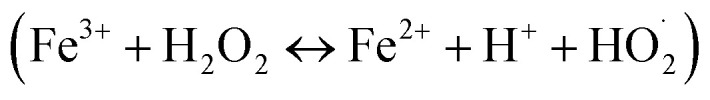
^56^ – a reducing agent can participate directly in the Cr(vi) reduction (*E*^o^(Fe^3+^/Fe^2+^) = 0.771 eV, *E*^o^(HCrO_4^−^_/Cr^3+^) = 1.350 eV, *E*^o^(Cr_2_O_7_^2−^/Cr^3+^) = 1.36 eV^57^).

Briefly, pH 6 was determined to be the optimal condition of MIL-88B((1 − *x*)Fe/*x*La)-NH_2_ (*x* = 0.010) for the photoreduction of Cr(vi) to Cr(iii). Subsequent experiments including assessing the photocatalytic ability of MIL-88B(Fe)–NH_2_ and La-doped MIL-88B(Fe)–NH_2_ photocatalysts and studying the photocatalytic kinetics and reusability of the catalyst would be conducted at this pH level.

**Fig. 9:**
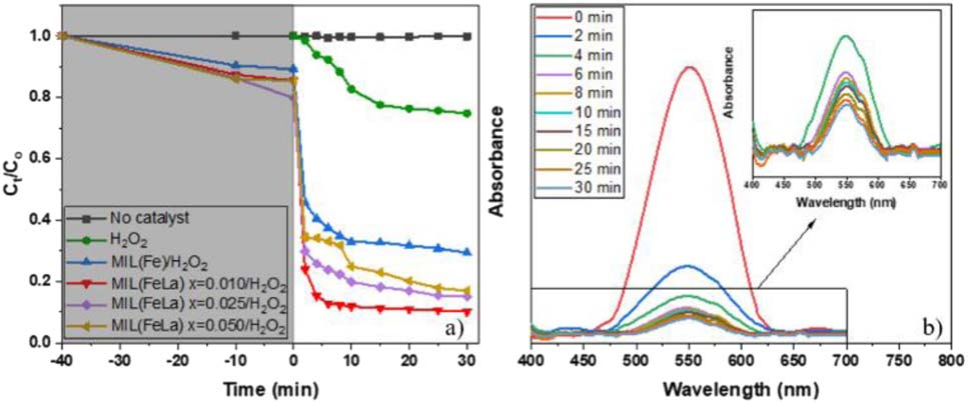
Cr(vi) photoreduction of various materials over time (a) and absorbance spectra of Cr–DPC solutions after photoreduction over MIL-88B((1 − *x*)Fe/*x*La)-NH_2_*x* = 0.010 (b) (initial Cr(vi) concentration: 20 ppm; pH = 6; catalyst dosage: 0.2 g L; H_2_O_2_: 3% (1 mL L^−1^); UV irradiation).

The photocatalytic activity of MIL-88B(Fe)–NH_2_ and MIL-88B((1 − *x*)Fe/*x*La)-NH_2_ (*x* = 0.010, 0.025, and 0.050) was evaluated by the degradation of 20 ppm Cr(vi) (pH = 6) with a catalyst dosage of 0.2 g L^−1^ in the presence of 3% H_2_O_2_ (1 mL L^−1^) under UV irradiation. Control tests were also performed using: (i) only Cr(vi) solution (without catalysts and H_2_O_2_), and (ii) Cr(vi) solution with only H_2_O_2_. As shown in Fig. 9a, there is no change in Cr(vi) concentration in the control test (i) and the Cr(vi) concentration change in the test (ii) was relatively low (approximately 25.2% after 30 minutes of UV irradiation). Meanwhile, the Cr(vi) reduction of MIL-88B–NH_2_ photocatalysts occurs rapidly, especially in first 2 minutes. The La-doped MIL-88B(Fe)–NH_2_ materials exhibit a better catalytic ability, with efficiencies reaching 88.21, 81.19 and 80.26% for MIL-88B((1 − *x*)Fe/*x*La)-NH_2_ with *x* = 0.010, 0.025 and 0.050, respectively, than that of MIL-88B(Fe)–NH_2_, with an efficiency of 67.08, after 30 minutes of irradiation. Obviously, the performance is improved when using La-doped MIL-88B(Fe)–NH_2_ catalysts. Introducing a small amount of La^3+^ into various nanomaterials such as MOFs, COFs, perovskites and semiconductor materials has been demonstrated to slow down the e^−^/h^+^ recombination rate, thereby enhancing the catalytic activity.^30,58,59^ As such, the La^3+^ appearance in the MIL-88B–NH_2_ structure could inhibit the e^−^/h^+^ recombination process, and the Cr(vi) photocatalytic efficiency here could be decided by the e^−^/h^+^ recombination rate.

Moreover, the highest amount of Cr(vi) is reduced when using MIL-88B((1 − *x*)Fe/*x*La)-NH_2_ with *x* = 0.010 (88.21%), and a slight decreasing trend is recorded when the La^3+^ content increases, 81.19% for MIL-88B((1 − *x*)Fe/*x*La)-NH_2_ (*x* = 0.025) and 80.26% for MIL-88B((1 − *x*)Fe/*x*La)-NH_2_ (*x* = 0.050). In comparison with the *E*_g_ values of MIL-88B(Fe/La)–NH_2_ materials (2.00 eV at *x* = 0.010; 2.23 eV at *x* = 0.025; and 2.41 eV at *x* = 0.050), the catalytic performance of the material with a higher bandgap energy is lower. Therefore, the performance is impacted partially by the increase in the bandgap energy.

From the above-mentioned findings, MIL-88B((1 − *x*)Fe/*x*La)-NH_2_ (*x* = 0.010) is selected to continue further research on the photoreduction process of Cr(vi) to Cr(iii) in water.

**Fig. 10:**
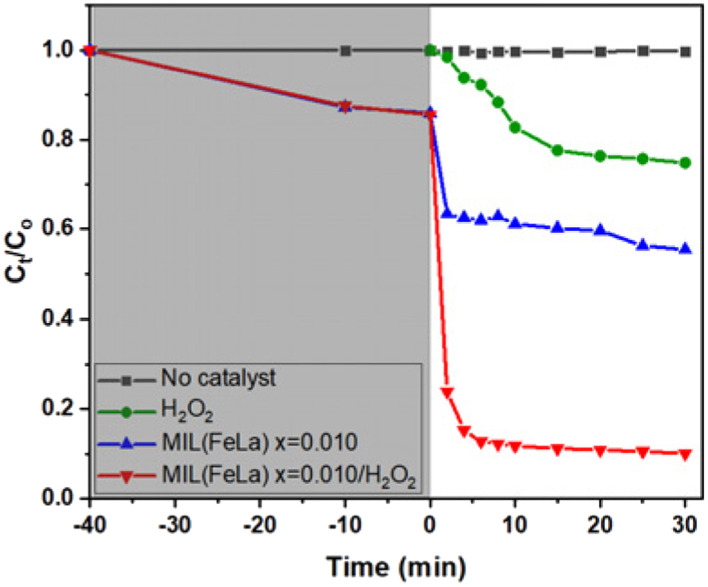
Cr(vi) photoreduction over time of H_2_O_2_, MIL-88B((1 − *x*)Fe/*x*La)-NH_2_ (*x* = 0.010) (without/with H_2_O_2_).

To evaluate the influence of H_2_O_2_ on the photocatalytic performance, another control experiment was performed: Cr(vi) degradation using photocatalyst MIL-88B((1 − *x*)Fe/*x*La)-NH_2_ (*x* = 0.010) without H_2_O_2_ (pH = 6). The obtained efficiency is only about 35%, considerably lower than that achieved when using the photocatalyst in the presence of H_2_O_2_ (Fig. 10). Apparently, H_2_O_2_ plays an important role in the photoreduction process of Cr(vi) to Cr(iii). It helps e^−^/h^+^ separation to be more fruitful thanks to combining with e^−^ (e^−^ + H_2_O_2_ → ˙OH + OH^−^),^20^ thereby limiting the ability to recombine e^−^/h^+^ and promoting the conversion process. In addition, the conversion performance is also enhanced by the production of reducing agent Fe^2+^*via* the reaction of Fe^3+^ and 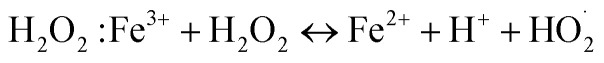.^56^

**Fig. 11:**
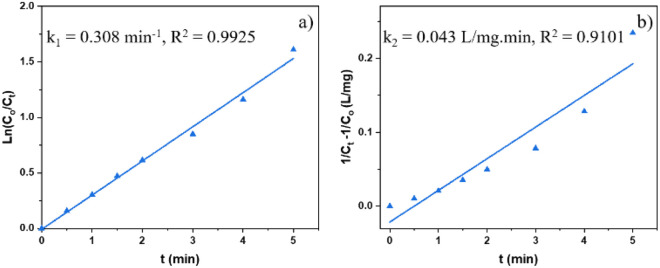
Pseudo-1st- (a) and pseudo-2nd-order (b) kinetic models for the photocatalysis process of MIL-88B((1 − *x*)Fe/*x*La)-NH_2_ (*x* = 0.010) (dosage: 0.2 g L^−1^).

In terms of kinetics, pseudo-first-order (1.1) and pseudo-second-order (1.2) kinetic models were used to study the kinetics of the photocatalytic reaction of Cr(vi) reduction on MIL-88B((1 − *x*)Fe/*x*La)-NH_2_ (*x* = 0.010) for first five minutes. The calculated results show that the photocatalytic reaction obeys the pseudo-first-order model with a rate constant *k*_1_ = 0.308 min^−1^ (*R*^2^ = 0.9925) (Fig. 11). The MIL-88B((1 − *x*)Fe/*x*La)-NH_2_ (*x* = 0.010) material exhibits a significantly higher rate constant than that of some other MOFs such as NH_2_-ZIF-8 (0.0057 min^−1^),^60^ NH_2_-MIL-88B(Fe)/CD-50 (0.0220 min^−1^),^19^ NNU-36 (0.0468 min^−1^)^61^ and MIL-53(Fe) (0.1154 min^−1^),^52^ suggesting its high efficiency.

1.1 In(*C*_0_/*C*_*t*_ = *kt*)

1.2
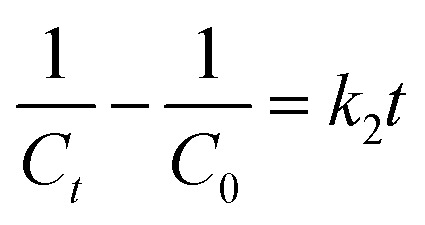
where *t* is the reaction time (min); *C*_0_ and *C* correspond to initial and remaining concentrations of Cr(vi) (mg L^−1^); *k*_1_ (min^−1^) and *k*_2_ (L mg^−1^ min^−1^) correspond to rate constants of the pseudo-1st- and 2nd-order models.

**Fig. 12:**
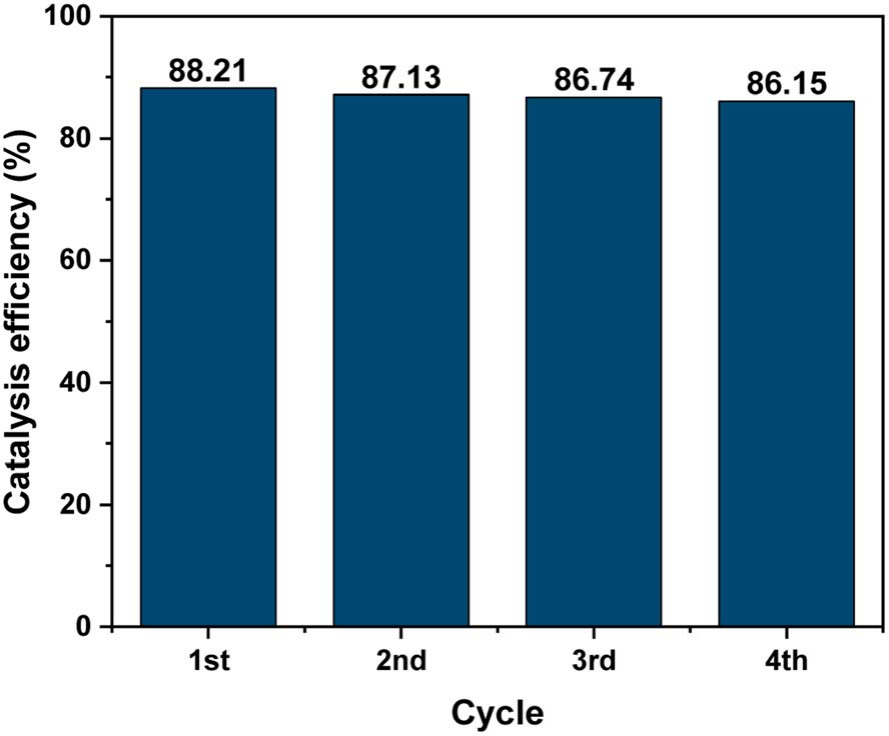
Reusability of MIL-88B((1 − *x*)Fe/*x*La)-NH_2_ (*x* = 0.010).

The stability of the MIL-88B((1 − *x*)Fe/*x*La)-NH_2_ (*x* = 0.010) photocatalyst was tested by recovering and reusing 4 times. Obviously, the photodegradation of Cr(vi) remains relatively stable in the subsequent runs, as shown in Fig. 12, indicating the high stability of the material. It can be seen that MIL-88B((1 − *x*)Fe/*x*La)-NH_2_ (*x* = 0.010) shows good photocatalytic ability with high performance and high stability in comparison to previous works.^19,53,60^

**Fig. 13:**
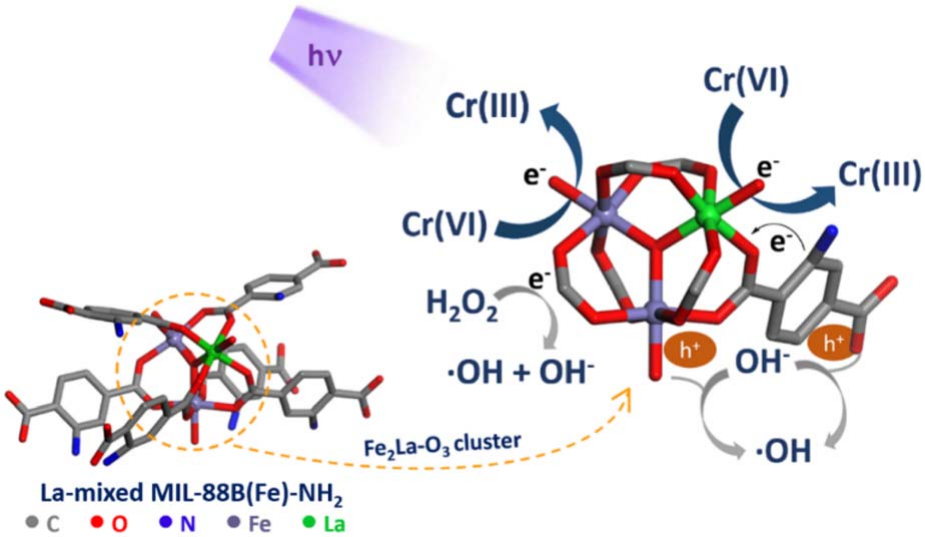
Proposed mechanism of Cr(vi) photoreduction onto La-doped MIL-88B(Fe)–NH_2_.

MIL-88B(Fe)–NH_2_ was reported to be an effective photocatalyst in the reduction of aqueous Cr(vi) thanks to the LMCT mechanism.^62,63^ Based on this, the proposed mechanism of MIL-88B((1 − *x*)Fe/*x*La)-NH_2_ (*x* = 0.010) for the photoreduction of Cr(vi) to Cr(iii) in water is described as follows (Fig. 13): under UV irradiation, both the NH_2_-TPA ligand and the Fe_3–*n*_La_*n*_O cluster (*n* = 0, 1, 2, and 3) are excited. Fe_3–*n*_La_*n*_O clusters generate e^−^/h^+^ pairs by absorbing photon energy, and electrons move to the conduction band leaving holes in the valence band (reaction (1)). The photoexcited electrons in the ligand move to the ion metal cluster, and this path is promoted more by amino groups. These generated electrons mainly participate in the reduction of Cr(vi) to Cr(iii) (reaction (2)). The probability of e^−^/h^+^ recombination is minimized thanks to the e^−^ trapping ability of H_2_O_2_ (reaction (3)), and the h^+^ trapping ability of OH^−^ ions (generated from reaction (3)^64^) and H_2_O molecules (reactions (4) and (5)). Furthermore, Fe^2+^ ions produced from the Fenton reaction and ˙O_2_^−^ radicals produced from oxygen reduction by photoelectrons also contribute to Cr(vi) reduction (reactions (6–10)).

1 La-doped MIL-88B(Fe)–NH_2_ + *hν* → e^−^ + h^+^

2 Cr(vi) + 3e^−^ → Cr(iii)

3 e^−^ + H_2_O_2_ → ˙OH + OH^−^

4 h^+^ + OH^−^ → ˙OH

5 2h^+^ + 2H_2_O → O_2_ + 4H^+^

6
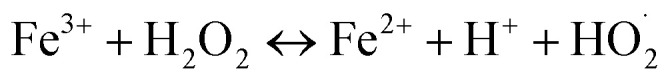

7 3Fe^2+^ + Cr(vi) → 3Fe^3+^ + Cr(iii)

8 O_2_ + e^−^ → ˙O_2_^−^

9 ˙O_2_^−^ + Cr(vi) → Cr(v) + O_2_

10 Cr(v) + 2e^−^ → Cr(iii)

## Experimental

### Chemicals and instrumentation

Iron(iii) chloride hexahydrate (FeCl_3_·6H_2_O; 99% wt), lanthanum(iii) nitrate hexahydrate (La(NO_3_)_3_·6H_2_O; 98% wt), 2-aminoterephthalic acid (H_2_N–C_6_H_3_-1,4-(COOH)_2_ (NH_2_-TPA); 99% wt), methanol (CH_3_OH (MeOH); 99.8% v/v), ethanol (C_2_H_5_OH (EtOH); 99.5% v/v), potassium dichromate (K_2_Cr_2_O_7_; 99.8% wt), 1,5-diphenylcarbazide ((C_6_H_5_NHNH)_2_CO (DPC); 98% wt), sodium hydroxide (NaOH; 97% wt) and hydrochloric acid (HCl; 37% wt) were purchased from Sigma-Aldrich. Dimethylformamide (C_3_H_7_NO (DMF); 99.94% v/v) was purchased from Fisher Chemical.

A Siemens D5005 diffractometer (Cu-K_α_ radiation, *λ* = 1.54056 Å), a Hitachi S4800 scanning electron microscope, an ISIS 300 energy-dispersive X-ray spectrometer, a Gemini VII 2390 surface analyzer, a NICOLET iS50FT-IR spectrometer and a V-750 UV-visible spectrophotometer were used to perform XRD, SEM, EDS, BET, FT-IR and UV-vis DRS measurements, respectively. An Agilent 8453 UV-visible spectroscopy system was used to support for the determination of Cr(vi) concentrations.

### Synthesis of La-doped MIL-88B(Fe)–NH_2_

The fabrication process of La-doped MIL-88B(Fe)–NH_2_ was referred from that of MIL-88B(Fe)–NH_2_, as reported in our previous work.^16^ In particular, MIL-88B((1 − *x*)Fe/*x*La)-NH_2_ materials were synthesized by an one-pot solvothermal method using a DMF solvent with a fixed molar ratio of H_2_N–C_6_H_3_-1,4-(COOH)_2_ (NH_2_-TPA) ligand to metal ions of 1.5, under reaction conditions of 150 °C and 12 hours (*x*: the molar ratio of La^3+^ to the molar total of the metal ion, 
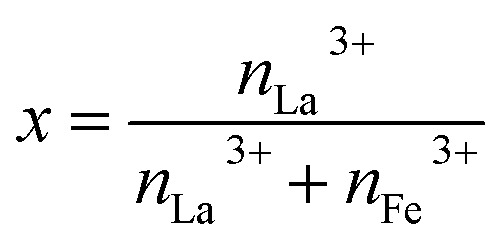
). An appropriate amount of FeCl_3_·6H_2_O and La(NO_3_)_3_·6H_2_O, and 0.6268 g NH_2_-TPA were dissolved into 50 mL DMF so that *x* reaches values of 0.010, 0.025, 0.050 and 0.10. The obtained solutions were sealed in autoclaves and then heated at 150 °C within 12 hours. Afterward, solid products were washed with DMF, methanol and distilled water and subsequently dried in a vacuum dryer. The fabricated MIL-88B((1 − *x*)Fe/*x*La)-NH_2_ materials are in a brown-colored powder form.

### Photocatalytic experiments

The photocatalytic performance of La-doped MIL-88B(Fe)–NH_2_ materials was studied through the photocatalytic reduction of Cr(vi) using a Hg lamp (250 W) as an ultraviolet light source.

The photoreduction efficiency of MIL-88B((1 − *x*)Fe/*x*La)-NH_2_ (*x* = 0.010) towards aqueous Cr(vi) solutions with different pH values was evaluated to study the influence of pH environment on the material's photocatalysis activity. The investigated pH values were in the range of 2–8, adjusted by HCl and NaOH. Particularly, the photocatalyst was dispersed in the Cr(vi)-containing solution (initial Cr(vi) concentration: 20 ppm; photocatalyst dosage: 0.2 g L^−1^) in a glass beaker in the darkness until an adsorption–desorption equilibrium was reached. Next, 3% H_2_O_2_ solution was added into the reaction system (1 mL L^−1^), and illuminated at the same time. Subsequently, the mixture was collected at determined intervals, and the catalyst was separated by centrifugation. The Cr(vi) concentration was then determined by a diphenylcarbazide method. In this method, Cr(vi) ions react with 1,5-diphenylcarbazide (DPC) ligands to form a purple-coloured complex (Cr–DPC complex) under the acidic condition.^51,65^

After finding out the optimal pH condition, similar experiments were performed to assess the Cr(vi) photoreduction ability of MIL-88B(Fe)–NH_2_ and MIL-88B((1 − *x*)Fe/*x*La)-NH_2_ (*x* = 0.010, 0.025, and 0.050) materials, impacts of H_2_O_2_ appearance on the Cr(vi) photoreduction, photocatalytic kinetics and reusability of the photocatalyst.

## Conclusions

In summary, a series of MIL-88B((1 − *x*)Fe/*x*La)-NH_2_ materials have been synthesized *via* a one-pot solvothermal approach and characterized by various measurement techniques including XRD, SEM, SEM-EDS, BET analysis, FT-IR spectroscopy and UV-vis DRS. The results indicate that MIL-88B((1 − *x*)Fe/*x*La)-NH_2_ materials were fabricated successfully at *x* = 0.010, 0.025 and 0.050. MIL-88B((1 − *x*)Fe/*x*La)-NH_2_ (*x* = 0.010, 0.025 and 0.050) materials were then used as photocatalysts for the aqueous Cr(vi) reduction. Compared to pristine MIL-88B(Fe)–NH_2_, La-doped MIL-88B(Fe)–NH_2_ materials display a better photocatalytic efficiency, and the best is achieved on MIL-88B((1 − *x*)Fe/*x*La)-NH_2_ (*x* = 0.010). In addition, the impact of the pH environment on the reduction performance of Cr(vi), the photocatalytic kinetics and reusability of this catalyst were studied. The output shows that the kinetics of photocatalytic reaction follows the pseudo-1st-order model, and the material exhibits high efficiency under the weak acidic condition and high stability after 4 running cycles. The harvested knowledge in this work is expected to contribute to the development of mixed-MOFs in the catalysis area for wastewater treatment.

## Data availability

Data for this article, including SEM, EDX, XRD, BET surface area, FTIR, and photocatalytic results are available at Open Science Framework at https://osf.io/me67s.

## Author contributions

MHDT, LGDT: investigation, data collection, writing – original draft preparation. HPNT: resources, reviewing and editing; DCH, DDL: writing – reviewing and editing. DDL, MHDT: visualization, editing, funding acquisition & supervision. All authors approved the manuscript.

## Conflicts of interest

The authors declare that they have no known competing financial interests or personal relationships that could have appeared to influence the work reported in this paper.
